# Clonality in haematopoietic stem cell ageing

**DOI:** 10.1016/j.mad.2020.111279

**Published:** 2020-07

**Authors:** Maria Terradas-Terradas, Neil A. Robertson, Tamir Chandra, Kristina Kirschner

**Affiliations:** a Institute of Cancer Sciences, University of Glasgow, Glasgow G61 1BD, UK; b MRC Human Genetics Unit, University of Edinburgh, Edinburgh, EH4 2XU, UK

**Keywords:** Clonal haematopoiesis of indeterminate potential, Environment, Cell-Intrinsic, Ageing, *DNMT3A*, *TET2*

## Abstract

Clonal haematopoiesis of indeterminate potential (CHIP) is widespread in the elderly. CHIP is driven by somatic mutations in leukaemia driver genes, such as Janus Kinase 2 (*JAK2*), Tet methylcytosine dioxygenase 2 (*TET2*), ASXL Transcriptional Regulator 1 (*ASXL1*) and DNA (cytosine-5)-methyltransferase 3A (*DNMT3A*), leading to reduced diversity of the blood pool. CHIP carries an increased risk for leukaemia and cardiovascular disease. Apart from mutations driving CHIP, environmental factors such as chemokines and cytokines have been implicated in age-dependent multimorbidities associated with CHIP. However, the mechanism of CHIP onset and the relationship with environmental and cell-intrinsic factors remain poorly understood. Here we contrast cell-intrinsic and environmental factors involved in CHIP development and disease propagation.

## Highlights

• Somatic driver mutations lead to clonal haematopoiesis of indeterminate potential (CHIP) in aged haematopoietic stem cells.
• CHIP is associated with a variety of age-related multimorbidities.
• How environmental and cell-intrinsic factors contribute to CHIP and development of multimorbidities is poorly understood.
• Increased inflammatory signalling with age might be one mechanism driving age-related disease and favouring outgrowth of HSCs carrying specific driver mutations.

## 1 Introduction

Age is the single most significant factor underlying the onset of many haematological malignancies (de Magalhães, 2013), with changes in the clonal composition towards a myeloid bias commonly occurring with advanced age (Cho et al., 2008). The onset of clonal haematopoiesis of indeterminate potential (CHIP) in the haematopoietic stem and progenitor cell (HSPC) compartment is also associated with haematological malignancies (Genovese et al., 2014). CHIP is apparent in the general population from age 60 with a steady increase in prevalence to 18–20% of individuals aged over 90 years at 2% variant allele frequency (VAF) (McKerrell et al., 2015). CHIP is driven by somatic mutations in leukaemic driver genes, thereby reducing the diversity of the stem cell pool. Epigenetic modifiers such as *TET2*, *ASXL1* and *DNMT3A* are the most frequently mutated genes in CHIP. *TET2* and *DNMT3A* are epigenetic regulators involved in DNA methylation impacting self-renewal and differentiation capacities of haematopoietic stem cells (HSCs) while *ASXL1* - a member of the polycomb repressive complex - is involved in chromatin remodelling and affects hematopoietic repopulating capacity and expansion of the haematopoietic stem cell compartment (Bowman et al., 2018; Challen et al., 2011; Fuster et al., 2017; Jeong et al., 2018; Ko et al., 2011; Li et al., 2011; Lu et al., 2016; Mayle et al., 2015; Moran-Crusio et al., 2011; Pan et al., 2017; Quivoron et al., 2011; Wang et al., 2014). Interestingly, one hallmark of ageing is the global loss of methylation and profound changes to heterochromatin (Chandra and Kirschner, 2016; Cypris et al., 2019).

*JAK2V617F* is a common synonymous variant that is frequently mutated in CHIP and age-related myeloid malignancies (Chen et al., 2012), where the *JAK2* tyrosine phosphatase is constitutively activated driving a plethora of downstream pathways such as the phosphoinositide-3-kinase/Protein kinase B pathway (PI3K/AKT), Signal Transducers and Activators of Transcription (STAT) and RAS/RAF/MEK/ERK Mitogen-activated protein kinase pathways. Together these pathways confer a proliferative advantage, resistance to DNA damage mediated apoptosis and can activate an inflammatory response (Chen et al., 2012).

DNA damage response and stress-related genes Tumour Suppressor 53 (*TP53*) and Protein Phosphatase, Mg2+/Mn2+ Dependent 1D (*PPM1D*) are another class of mutations identified in CHIP (Genovese et al., 2014; McKerrell et al., 2015). *TP53* and *PPM1D* mutations are predominantly mutated in leukocytes of patients who have undergone cancer treatment for solid tumours and display clonal haematopoiesis (Coombs et al., 2017), associating genotoxic stress with clonal selection. In this study, clonal haematopoiesis was associated with secondary leukaemia development following solid cancer therapy. In other studies elucidating the mechanism of mutant *PPM1D* in clonal haematopoiesis and subsequent therapy-related myeloid malignancies (Hsu et al., 2018; Kahn et al., 2018), mutant *PPM1D* seemed to confer resistance to apoptosis in the context of genotoxic stress. Therapy-induced senescence is a prominent feature in cancer therapy and an alternative mechanism of cancer therapy resistance, with *TP53* being one of the most prominent senescence inducers, engaging a specific, downstream senescence programme that differs profoundly from apoptosis (Kahlem et al., 2004; Kirschner et al., 2015). Whether *TP53* mutations shift the pathway away from apoptosis towards senescence in the context of CHIP remains to be elucidated.

Lastly, splicing factors emerge late in the pathogenesis of CHIP with the most prominent being Splicing Factor 3b Subunit (*SF3B1*), *SRSF2* (Serine and Arginine Rich Splicing Factor 2) and U2 Small Nuclear RNA Auxiliary Factor 1 (*U2AF1*) (Inoue et al., 2016). These mutations alter RNA splicing in a sequence-specific manner and might affect downstream pathways leading to CHIP over a longer period of time.

CHIP is associated with an increased risk for haematological cancers and all-cause mortality, specifically coronary heart disease and ischaemic stroke, for which age is a major risk factor (Genovese et al., 2014; Jaiswal et al., 2017, 2014; McKerrell et al., 2015; Zink et al., 2017). In addition, CHIP in patients with heart failure results in increased mortality and hypertension (Dorsheimer et al., 2019). In this review, we will discuss factors that can lead to the onset of CHIP and its age-associated diseases contrasting fitness acquired by mutations (cell-intrinsic) against cell-extrinsic changes in the ageing environment.

## 2 Cell-intrinsic contributions to CHIP

**Fig. 1:**
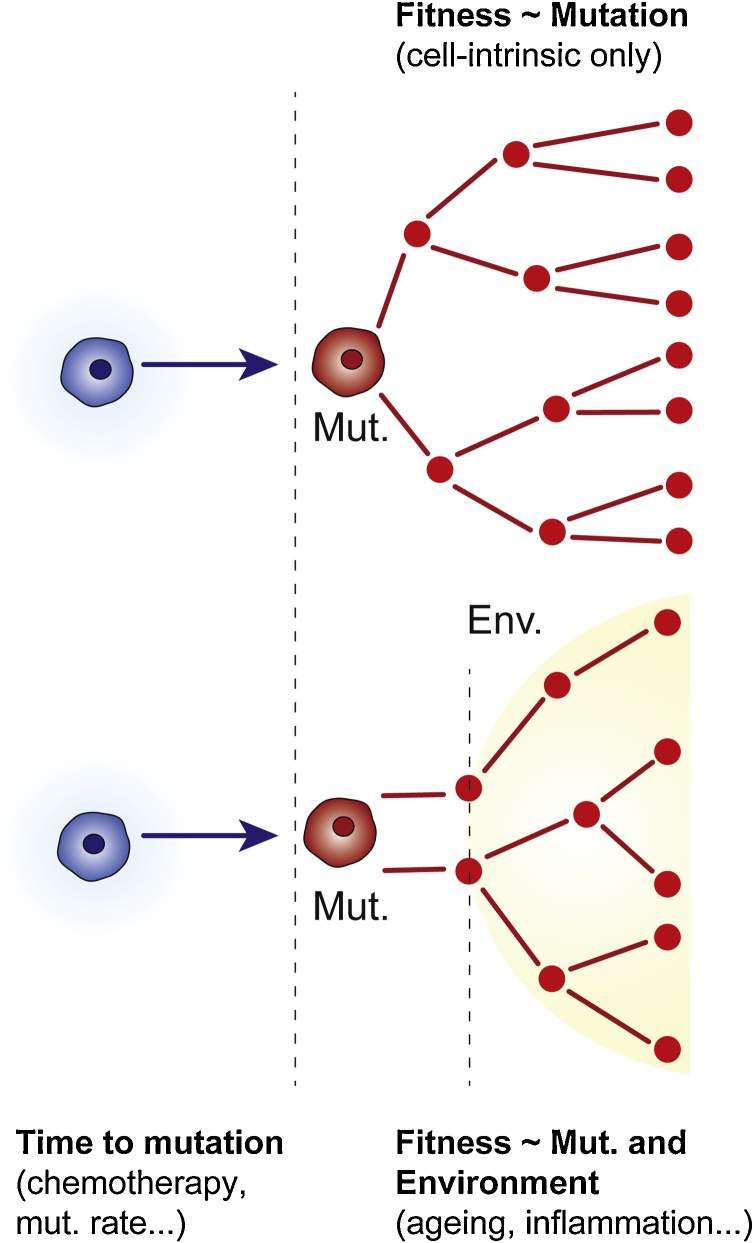
**Is CHIP dependent on the environment or driven by cell intrinsic factors?** Upper panel: **CHIP is driven cell-intrinsically.** Here, the time to acquisition of the CHIP mutation (Mut.) and the change in fitness conferred by the mutation are the determining factors of clonal outgrowth. Average time to mutation depends on a variety of factors, including sequence context of the mutation, mutation rate and genotoxic events, such as chemotherapy. Lower Panel: **CHIP is driven through cell-intrinsic and environmental factors**. Here, the time to acquisition of the CHIP mutation (Mut.) and the change in fitness conferred by the mutation are again determining factors. However, clonal outgrowth is enhanced or enabled by environmental changes (Env. yellow background). Supposed environmental factors are discussed in the main text and include inflammation and other age-related changes.

Several landmark studies reported the occurrence of CHIP in healthy aged individuals using various deep sequencing approaches of peripheral blood mononuclear cells (PBMCs). The major driver mutations, such as *JAK2*, *TET2* and *DNMT3A,* in all cohorts examined overlap, with varying allele frequencies of the distinct driver mutations. A few genes were only reported in some cohorts, such as the DNA damage response pathway gene *PPM1D* (Genovese et al., 2014; Jaiswal et al., 2017, 2014; McKerrell et al., 2015). The occurrence of CHIP mutations with age pointed to a time-dependent acquisition pattern, leading to a competitive advantage and driving CHIP with advanced age (Fig. 1). This is especially true when considering splicing factors.

Mouse studies of commonly mutated CHIP genes support the notion that clonal outgrowth is driven by cell-intrinsic properties in the HSPC compartment. Among the most frequently mutated genes is *Dnmt3a*. Challen and colleagues demonstrated that conditional loss of *Dnmt3a* in HSCs impairs their differential potential by altering DNA methylation (Challen et al., 2011). In knockout mice, loss of *Dnmt3a* immortalises HSCs (Jeong et al., 2018), leading to skewed division potential with HSCs being primed towards self-renewal for up to twelve rounds of transplantation with gradual and focal loss of DNA methylation at key HSC self-renewal sites. In this study, however, transformation required additional mutations (Jeong et al., 2018). In a study by Mayle et al., transplantation of murine *Dnmt3a*-knockout HSCs into irradiated wild-type mice resulted in the development of a range of haematological malignancies, leading to increased mortality. These results suggest a cell-intrinsic role of *Dnmt3a* loss in HSCs in acquiring a preleukemic state (Mayle et al., 2015). This is in accordance with another mouse study, where HSPCs with a *DNMT3aR882H* mutation, the most commonly found CHIP mutation, promotes leukaemia only in the presence of other oncogenes such as N-RasG12D (Lu et al., 2016). *DNMT3aR882H* alone led to hypomethylation at cis-elements of essential stemness genes such as the Meis Homeobox 1 (*Meis1)*, MN1 proto-oncogene (*Mn1*), and Hoxa gene clusters, and led to increased expression of a panel of stemness genes (Lu et al., 2016).

*TET2* is also commonly mutated in individuals with CHIP. Several studies using *Tet2* knockout and mutant mouse models have explored cell-intrinsic mechanisms of HSCs leading to malignant transformation (Ko et al., 2011; Moran-Crusio et al., 2011; Quivoron et al., 2011). All these studies showed an expansion of the HSC compartment, as well as enhanced self-renewal potential associated with TET2 loss-of-function. Interestingly, Li and colleagues demonstrated an increase of the murine HSC pool in *Tet2* knockout mice, with only a subset of mice developing myeloid malignancies (Li et al., 2011). A later study showed spontaneous development of different haematological malignancies in *Tet2* knockout mice, resulting from increased mutagenicity (Pan et al., 2017). Single-cell -targeted sequencing revealed a higher mutation rate in *Tet2*-/- HSPCs particularly at sites which gained 5-hydroxymethylcytosine, suggesting TET2 mediated cell-intrinsic changes in HSPCs leading to malignant transformation (Pan et al., 2017).

It is well documented that CHIP mutations can result in inflammation, leading to, for example, enhanced atherosclerosis. In this context, Jaiswal and colleagues (Jaiswal et al., 2014) showed that, when atherosclerosis prone mice were transplanted with *Tet2* knockout bone marrow cells, the development of atherosclerosis was markedly accelerated on the background of a high cholesterol, high-fat diet. In addition, higher levels of pro-inflammatory chemokines could be detected in the serum of these mice. On the molecular level, *Tet2* knockout macrophages, when cultured with low-density lipoprotein, displayed a highly inflammatory transcriptional signature compared to wild type (WT) macrophages, suggesting the involvement of inflammatory signalling in the progression of atherosclerosis on a *Tet2* mutant background. In addition, Fuster and colleagues studied the effects of *Tet2* mutant HSPCs and their progeny in atherosclerosis prone mice deficient in low-density lipoprotein receptor (*Ldlr*–/–) by competitive transplantation (Fuster et al., 2017). The authors demonstrated that *TET2* loss of function in macrophages exacerbated NLR Family Pyrin Domain Containing 3 (*Nlrp3*) mediated Interleukin 1 beta (IL-1b) production which in turn accelerated atherosclerosis in a context of CHIP, thereby demonstrating that CHIP leads to increased inflammation. Inflammation resulting from mutations in CHIP associated genes is further evidenced by a mouse study, examining the co-operating oncogenic effects of *Jak2V617F* and *Dnmt3a* in HSPCs (Jacquelin et al., 2018). *Dnmt3a* loss on top of the *Jak2V617F* mutation led to the activation of inflammatory signalling, inducing myelofibrosis. A recent study showed that the *Jak2V617F* mutation in HSPCs gave rise to circulating myeloid cells with enhanced pro-inflammatory properties on its own in mouse models of cardiac injury (Sano et al., 2019). These studies demonstrate a role for cell-intrinsic activation of inflammatory pathways as a consequence of CHIP.

Modelling approaches contribute further to evidence for mutations alone being able to explain CHIP. Watson and colleagues (Watson et al., 2020) used PBMC sequencing data from various CHIP studies, analysing 50,000 individuals at varying VAFs. They showed that modelling clone size distributions based on a change of fitness conferred by driver mutations and the probability (time) to acquire these mutations was enough to predict the observed distributions from the collected data sets.

## 3 Contributing environmental factors towards CHIP

Systemic inflammation from the environment can promote CHIP through, for example, short term inflammatory stress caused by lipopolysaccharides in HSPCs (Cai et al., 2018) (Fig. 1). Murine *Tet2* knockout HSPCs display a survival advantage compared to WT HPSCs during acute inflammation. Following inflammation, *Tet2* knockout HSPCs activated the interleukin 6 (Il6) mediated Stat3/Morbid axis, leading to the upregulation of B-cell lymphoma 2 (*Bcl2*) pro-survival factor and reduced apoptosis in these cells. Therefore, cell-extrinsic factors such as an inflammatory milieu can enhance the competitive fitness of CHIP mutant HSPCs over time.

DNA damage accumulates in aged HSCs and leads to decreased stem cell function. One study linked increased DNA damage directly to exit from the homeostatic quiescent state of HSCs as a response to physiological stresses, explaining the accumulation of DNA damage in aged HSC (Walter et al., 2015). The authors used polyinosinic:polycytidylic acid to mimic viral infections, effectively mounting a type I interferon response (Walter et al., 2015). In addition, a recent study implicated Rad21/Cohesin mediated NFkB signalling in aged HSCs with loss of self-renewal in favour of myeloid biased differentiation in response to inflammatory stimuli (Chen et al., 2019). Inflammation-induced exit from quiescence and ageing-associated inflammation in blood serum and tissue could, therefore, influence the selection of mutant HSPCs carrying CHIP mutations.

A small study in 187 ulcerative colitis (UC) patients, an autoimmune disease characterised by increased levels of proinflammatory cytokines with an average onset age before 30 years, analysed targeted PBMC sequencing for CHIP mutations (Zhang et al., 2019). Albeit patient numbers being small and the lack of a validation cohort, the study revealed *DNMT3A* and *PPM1D* as the most prevalent mutations with a lower incidence of *TET2* mutations. In this cohort, overall CHIP was slightly higher in UC patients, with *DNMT3A* mutant patients revealing significantly higher levels of serum interferon-gamma (IFNg), but not tumour necrosis factor-alpha. Interestingly, *DNMT3A* VAF was a significant contributor to increased IFNg levels, suggesting that increased IFNg might select for *DNMT3A* mutations in UC. Given that the onset of UC mostly occurs before the age of 30 years - compared to the onset of CHIP occurring at least two decades later, this study might provide evidence of a pro-inflammatory milieu playing a role in CHIP onset (Ha et al., 2010). However, in a minority of UC patients, disease occurs at the age of 50 years or older. In this context, inflammation might occur first, resulting from, for example, *DNMT3A* mutant T-cell clones (Thomas et al., 2010) or other CHIP related inflammatory processes as discussed above.

## 4 Associations with CHIP

Most CHIP studies describe associations with age-related multimorbidities or ageing factors, leaving uncertainty over cell-intrinsic versus environmental factors and their contributions to CHIP.

A key determinant of CHIP is the presence of recurrent driver mutations that are functionally well described; however, many samples frequently present with not known causal variants - a phenomenon known as clonal haematopoiesis with unknown drivers (CH-UD) (Genovese et al., 2014). Genovese and colleagues performed whole-exome sequencing of PBMCs on 11,845 participants, where the majority had not known putative driver mutations, with 1333 participants displaying 1 mutation, 313 participants harbouring 2 mutations, and 272 with 3 to 18 somatic mutations in total (Genovese et al., 2014). The authors then defined CH-UD based on the mutational burden in passenger genes alone, rather than on the identity of the mutations. In some participants without known driver mutations, further analysis and deeper sequencing eventually revealed a candidate variant (Genovese et al., 2014), suggesting that detection sensitivity might have been limiting in the first instance. The absence of canonical CHIP variants might also be explained by copy-number alterations of affected genes; however, it is unlikely that these factors explain all cases. Indeed, some have hypothesised CH-UD may be linked to reduced HSC fitness with age which results in increased oligoclonality through a depletion of the HSC pool (Gibson and Steensma, 2018).

Robertson and colleagues (Robertson et al., 2019) recently showed an increase in epigenetic age, a correlate of biological age, in CHIP carriers when compared to individuals without detectable CHIP. In this study, CHIP mutations were annotated in the Lothian Birth Cohorts (LBCs) of 1921 (n = 550) and 1936 (n = 1091), two independent, longitudinal studies in the elderly using whole-genome sequencing. Epigenetic clock analysis was then performed on 450 K methylation arrays. Increased epigenetic age was noted when considering all CHIP mutations together, and *TET2* and *DNMT3A* mutations individually. These effects were larger than the known sex differences in age acceleration (male > female) in either cohort. Moreover, VAF was positively correlated with accelerated epigenetic age, suggesting a link to clone size, which could be driven by intrinsic or environmental factors.

Zink and colleagues performed deep whole-genome sequencing (WGS) in 11,262 Icelanders and identified 1403 cases of CHIP at 2% VAF, irrespective of driver mutation status (Zink et al., 2017). Overall CHIP in this cohort was much more common compared to other studies, with 50% of participants over the age of 85 being carriers, showing similar somatic mutation patterns as previously reported (*TET2*, *DNMT3A*, *ASXL1*, *PPM1D*). In this cohort, CHIP mutations were associated with reduced telomere length (Zink et al., 2017). This finding complements the notion that epigenetic age is altered in CHIP, suggesting that proliferation might be a feature contributing to cell-intrinsic ageing factors and systemic ageing. Interestingly, known driver mutations were only apparent in a fraction of CHIP carriers. Using modelling approaches, the authors suggested that some clones have arisen in the absence of mutations as a result of neutral drift, which would only act on a small number of active HSCs. However, the majority of CHIP cases in the absence of mutations remained unexplained, suggesting environmental influences.

Further evidence for clonal outgrowth due to increased age comes from a study where WGS of PBMCs from a 115-year-old woman was performed (Holstege et al., 2014). The authors detected 450 somatic mutations, which were reported as passenger mutations, leading to oligoclonal haematopoiesis (Holstege et al., 2014). The authors suggested that the finite lifespan of HSCs leads to CHIP rather than the acquisition of driver mutations. Whether HSC lifespan is mainly regulated by environmental (e.g. cytokines promoting proliferation) or cell-intrinsic factors (e.g. telomere length) remains to be seen. During ageing, senescence due to telomere shortening or other cues has been described as a main driver of a proinflammatory environment (Acosta et al., 2008; Coppé et al., 2008; Kuilman et al., 2008). Telomere shortening in bone marrow stromal cells was correlated with a dysfunctional haematopoietic environment and increased cytokines in ageing context (Ju et al., 2007). Whether senescence and SASP play a significant role during ageing of the blood compartment remains to be conclusively elucidated.

## 5 Concluding remarks

It is becoming increasingly clear that certain mutations lead to CHIP. For *TET2* and *SRSFP95H* mutations, myeloid bias was associated with CHIP or the initiation of myelodysplastic/myeloproliferative syndrome, whereas for *DNMT3A* multipotent stem cell origin was described in the context of CHIP (Buscarlet et al., 2018; Smeets et al., 2018). However, competition or cooperation between clones of distinct sizes, lineage origin and different types of mutations is currently unknown. For example, could an early clone create an environment favouring outgrowth of certain other clones over time, by, for example, promoting inflammation?

Although evidence is increasing for mutations driving CHIP, several studies also suggest clonal outgrowth can appear in the absence of driver mutations. One such scenario could be due to a selection based on transcriptional phenotypes in the absence of CHIP, as suggested for ageing mouse HSCs. In one study, only a minority subpopulation of HSCs developed transcriptional signatures commonly associated with HSC ageing such as *Tp53* (Kirschner et al., 2017). The majority of HSCs showed a transcriptome similar to that of young HSCs, suggesting heterogeneous ageing phenotypes on the transcriptional level (Kirschner et al., 2017). Whether proliferation of HSC subpopulations was driven cell-intrinsically, or whether HSC subpopulations were simply being kept in a low proliferative state over time, gaining an advantage over exhausted, pro-proliferative HSC populations with increased age, remains to be elucidated.

## Author contribution

K.K. and T.C. conceived the review. M.T.T., N.A.R., K.K. and T.C. wrote the manuscript.

## Declaration of Competing Interest

The authors declare no competing interests
